# Transcriptional inhibition by CDK7/9 inhibitor SNS-032 suppresses tumor growth and metastasis in esophageal squamous cell carcinoma

**DOI:** 10.1038/s41419-021-04344-w

**Published:** 2021-11-05

**Authors:** Huishan Zeng, Huiru Yang, Yifan Song, Dong Fang, Liang Chen, Zhijun Zhao, Chaojie Wang, Songqiang Xie

**Affiliations:** 1 School of Pharmacy, Henan University, N. Jinming Avenue, 475004 Kaifeng, Henan China; 2 The Key Laboratory of Natural Medicine and Immuno-Engineering, Henan University, N. Jinming Avenue, 475004 Kaifeng, China; 3 Department of Medicine and Therapeutics, Luohe Medical College, 462000 Luohe, China

**Keywords:** Targeted therapies, Targeted therapies

## Abstract

Metastasis is one of most lethal causes that confer a poor prognosis of patients with esophageal squamous cell carcinoma (ESCC), whereas there is no available target drug for metastatic ESCC currently. In this study, we aimed to determine whether the transcriptional inhibition by CDK7/9 inhibitor SNS-032 is activity against ESCC. MTT and soft agar assays were performed to examine the influence of SNS-032 on ESCC growth in vitro. Tumor xenograft in nude mice was used to assess the antitumor activity of SNS-032 in vivo. The roles of SNS-032 in ESCC metastasis were conducted by wound healing and transwell assays in vitro, and by a lung and a popliteal lymph node metastasis model in vivo. The results showed that CDK7 and CDK9 were highly expressed in ESCC cells; SNS-032 effectively inhibited cellular viability, abrogated anchorage-independent growth, and potentiated the sensitivity to cisplatin in ESCC cells in vitro and in vivo. In addition, SNS-032 induced a mitochondrial-dependent apoptosis of ESCC cells by reducing Mcl-1 transcription. SNS-032 also potently abrogated the abilities of ESCC cell migration and invasion through transcriptional downregulation of MMP-1. Importantly, SNS-032 remarkably inhibited the growth of ESCC xenograft, increased the overall survival, as well as diminished the lung and lymph node metastasis in nude mice. Taken together, our findings highlight that the CDK7/9 inhibitor SNS-032 is a promising therapeutic agent, and warrants a clinical trial for its efficacy in ESCC patients, even those with metastasis.

## Introduction

According to the GLOBOCAN estimates from the International Agency for Research on Cancer, esophageal cancer is the seventh most commonly diagnosed cancer, with an estimated 604,000 new cases, and is the sixth leading cause of cancer death (544,000 deaths) in 2020 [1]. As the most common histologic subtypes of esophageal cancer, esophageal squamous cell carcinoma (ESCC) is highly prevalent in the South-East and Central Asian region, especially in China, which contributes >50% of worldwide cases [1]. Although the diagnosis, staging, and therapeutic strategies for ESCC have improved in recent years, the prognosis remains poor. The overall 5-year survival rate in ESCC patients with stage III disease is about 10% and the median survival time in patients with stage IV disease is <1 year [2]. The high rates of local invasion and regional lymph node metastasis is the major detrimental event that leads to the poor prognosis of patients with ESCC, even after effective curative treatment [2, 3]. Therefore, it is urgently need to find novel effective therapeutic drugs for the treatment of patients with metastatic ESCC.

Accumulating studies have demonstrated that malignant cells can be preferentially targeted by transcriptional inhibition, due to they are more reliant than normal cells on continuous expression of oncogenes [4]. Some members of the cyclin-dependent kinases (CDKs) family are associated with transcription control. In particular, CDK7 (also known as CDKN7) and CDK9 have major roles in promoting the initiation and elongation of transcription, respectively [5]. For instance, as an integral component of the transcription factor transcription factor II H, CDK7 facilitates transcription initiation by phosphorylating serines 5 and 7 (Ser5 and 7) in the heptad repeats of the C-terminal domain (CTD) of RNA polymerase II (Pol II) [6]. CDK9, as a portion of the positive transcription elongation factor-b, performs a complementary function by increasing the phosphorylation in the CTD of RNA Pol II at Ser2, which is required for transcript elongation [6, 7]. An immunohistochemical (IHC) analysis of 98 patients with ESCC showed that CDK7 was high expressed in 80 cases (81.63%). Additionally, elevated expression of CDK7 was positively associated with tumor grade and the poor prognosis. Moreover, silencing CDK7 by short hairpin RNA (shRNA) remarkably suppressed cell growth and increased chemotherapeutic sensitivity to cisplatin (CDDP) in ESCC cells [8]. Another study showed that CDK7 was highly expressed in 87/105 cases (82.86%) [9]. CDK7 was higher in ESCC tissues with lymph node metastases than in those without lymph node metastases. Moreover, CDK7 was positively correlated with E-cadherin, tumor grade, and tumor metastasis [9]. Additionally, pharmacological inhibition of CDK7 by selective inhibitor THZ1 possessed very strong antineoplastic activities against ESCC cells both in vitro and in vivo [10]. Although the expression of CDK9 in ESCC remains unclear, studies have demonstrated that CDK9 is overexpressed in many human malignant tumors and has been considered as an excellent target for drug development in cancer [11–14]. Indeed, selective genetic and pharmacological inhibition of CDK9 elicits a strong antineoplastic response in hematologic and solid tumor cells in vitro as well as in in vivo cancer models [15, 16]. Considering that both CDK7 and CDK9 play essential roles in regulation of transcription, therefore we speculated that dual targeting CDK7 and CDK9 may be a promising strategy to treat ESCC.

SNS-032 (formerly BMS-387032) was originally designed by Bristol-Myers Squibb Pharmaceutical Research Institute (Stamford, CT) in an effort to get a selective inhibitor against CDK2 [17]. Subsequent studies demonstrated that the antitumor activities of this compound primarily depend on interference with CDK7 and CDK9, with the IC_50_ values of 62 and 4 nM, respectively [5, 17, 18]. As a potent inhibitor of both CDK 7 and CDK9, SNS-032 has been demonstrated to effectively kill chronic lymphocytic leukemia (CLL) cells via blockage of phosphorylation of RNA polymerase II and inhibition of RNA synthesis in vitro regardless of prognostic indicators and treatment history [17]. The in vivo antitumor activity of SNS-032 was also demonstrated in mouse and human tumor models [5, 19, 20]. Importantly, a phase I study in heavily pretreated patients with CLL and multiple myeloma demonstrated that single-agent SNS-032 was well tolerated and showed mechanism-based target modulation as well as modest clinical activity [21]. Additionally, the data from a phase I clinical trial in patients with metastatic refractory solid tumors suggest that SNS-032 administered as a weekly 1-h infusion was well tolerated and may be feasible for oral administration, though the tolerability and bioavailability of the oral formulation would have to be formally assessed [22]. Whether SNS-032 has a potential role for the treatment of ESCC remains uncertain.

In this study, our purpose was to determine whether targeting CDK7/9 by SNS-032 is active against ESCC. We found that inhibition of transcription by SNS-032 potently inhibited ESCC cell proliferation, reduced colony formation, and triggered apoptosis both in vitro and in a nude mouse xenograft model. More importantly, SNS-032 effectively suppressed migration, invasion, lung metastasis, and lymph node metastasis in ESCC. These findings highlight that the CDK7/9 inhibitor SNS-032 is a promising antitumor agent against ESCC cells and warrants a clinical trial to further assess its efficacy in patients with ESCC, especially with metastasis.

## Results

### SNS-032 inhibits the growth of ESCC cells

**Fig. 1:**
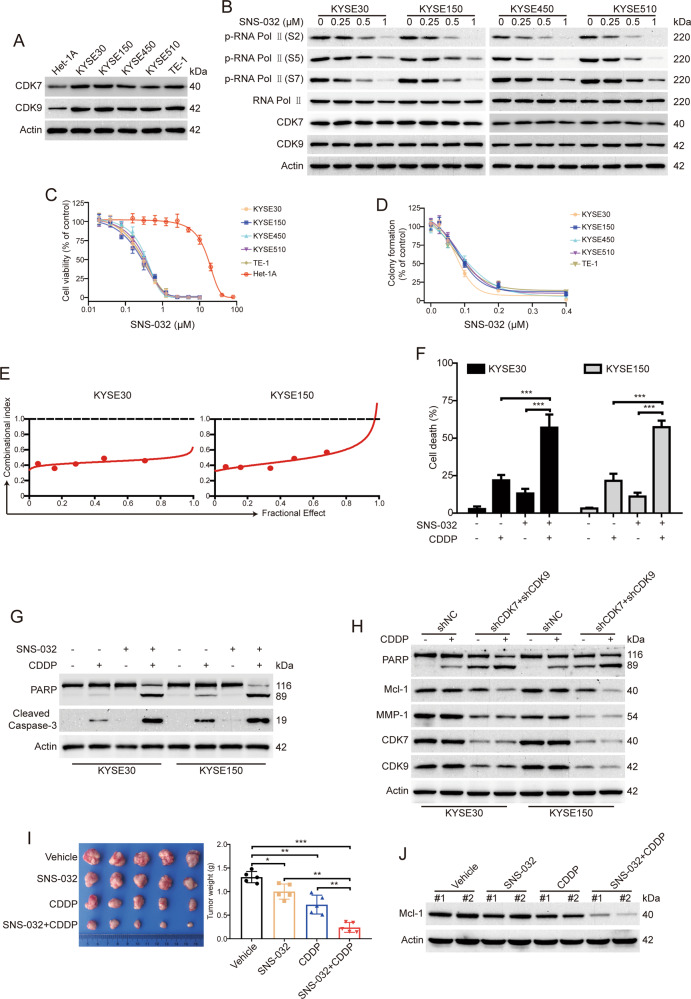
SNS-032 inhibits the growth of ESCC cells. **A** Western blot analysis was performed to detect the protein levels of CDK7 and 9 in ESCC cells and Het-1A cells. **B** SNS-032 inhibited CTD phosphorylation of RNA Pol II in ESCC cells. Cells were treated with increasing concentration of SNS-032 for 48 h; immunoblotting analysis with the indicated antibodies was performed. **C** SNS-032 selectively inhibited the cell viability of ESCC cells examined by MTT assay. Five kinds of ESCC cells (KYSE30, KYSE150, KYSE450, KYSE510, and TE-1) and one immortalized esophageal epithelial cell line (Het-1A) were incubated with escalating concentrations of SNS-032 for 72 h, and then the cell viability was examined by MTT assay. **D** SNS-032 inhibited the anchorage-independent growth of ESCC cells. **E** Synergistic effect of the combinational treatment between SNS-032 and CDDP in ESCC cells (KYSE30 and KYSE150) when cell viability was assessed by MTS assay. CI (combinational index) values <1 indicate synergism between the two drugs. **F**, **G** After incubation with SNS-032 (0.25 μM) in combination with CDDP (10 μM) for 48 h, cells were collected and subjected to trypan blue exclusion assay (**F**) and western blot analysis (**G**). + indicates the presence and − indicates the absence of the indicated drugs. ***P < 0.001, by one-way ANOVA with post hoc intergroup comparison with Tukey’s test. **H** Simultaneously silencing CDK7 and CDK9 enhanced apoptosis and downregulation of Mcl-1 induced by CDDP in ESCC cells. **I** Combined SNS-032 and CDDP treatment synergistically inhibits tumor growth of ESCC in vivo. KYSE30 cells in nude mice were treated with SNS-032 at 7.5 mg/kg, CDDP at 5 mg/kg, or both for 4 weeks, as described in “Materials and methods.” Representative images of tumors from each group of mice are shown, and tumor weight was measured at the end of the experiment. *P < 0.05, **P < 0.01, ***P < 0.001, one-way ANOVA with post hoc intergroup comparison by Tukey’s test. **J** Western blot analysis of Mcl-1 in tumors tissues derived from SNS-032, CDDP, or both treated mice.

To determine the inhibitory effects of SNS-032 on ESCC cells, we first examined the protein levels of CDK7 and CDK9 in ESCC cells by western blot analysis. As shown in Fig. 1A, the levels of CDK7 and CDK9 were much higher in all five tested ESCC cells (KYSE30, KYSE150, KYSE450, KYSE510, and TE-1) than those in Het-1A cells (immortalized esophageal epithelial cell line). Considering that SNS-032 is a selective inhibitor of CDK7 and CDK9 by blocking the phosphorylation of RNA Pol II at Ser 2, 5, and 7, we next examined whether SNS-032 inhibited the kinase activity of CDK7 and CDK9. As expected, SNS-032 remarkably downregulated the levels of phospho-RNA Pol II (Ser 2), phospho-RNA Pol II (Ser 5), and phospho-RNA Pol II (Ser 7), but not the total RNA Pol II, CDK7, and CDK9 in tested cell line of ESCC (Fig. 1B).

To ascertain the antineoplastic effects of SNS-032 on ESCC cells, the cell viability of Het-1A and five ESCC cell lines pretreated with increasing concentrations of SNS-032 for 72 h were detected by 3-[4,5-dimethylthiazol-2-yl]-2,5 diphenyl tetrazolium bromide (MTT) assay. As shown in Fig. 1C, the IC_50_ value in Het-1A cells was 16.84 μM, whereas the cell viability of five ESCC cell lines (KYSE30, KYSE150, KYSE450, KYSE510, and TE-1) were obviously suppressed in a dose-dependent manner by SNS-032, with IC_50_ values of 0.27, 0.25, 0.34, 0.32, and 0.28 μM, respectively, which was much lower than that in the immortalized esophageal epithelial cell line Het-1A. Additionally, silencing CDK7 or CDK9, but not CDK2, by shRNA made the KYSE30 and KYSE150 cells more insensitive to SNS-032 treatment when compared with the control group (Supplementary Fig. S1). We next performed soft agar assay to determine whether SNS-032 inhibits the anchorage-independent growth of ESCC cells. The results showed that SNS-032 drastically suppressed the clonogenicity of all tested ESCC cells in soft agar, with IC_50_ value ranging from 78.3 to 107.5 nM (Fig. 1D). Collectively, these data confirmed that SNS-032 effectively inhibited the tumor cell growth of ESCC in vitro.

### SNS-032 exhibits synergistic effect in ESCC when combined with CDDP

CDDP is the first-line chemotherapeutic drug for ESCC patients. We therefore examined whether SNS-032 has a synergistic effect with CDDP in ESCC cells. ESCC cells were exposed with SNS-032, CDDP, or SNS-032 and CDDP for 3 days, and then the cell viability was measured by MTT assay and the combined effect was analyzed according to the method of Chou and Talalay [23]. The results showed that there was a synergistic effect between SNS-032 and CDDP in inhibiting proliferation of the two tested ESCC cells (Fig. 1E). With another experiment, KYSE30 and KYSE150 cells were treated with SNS-032 (0.25 μM), CDDP (10 μM), alone or both for 48 h, the ratio of cell death was assessed by trypan blue exclusion assay. The results showed that either SNS-032 or CDDP induced minimal lethality of ESCC cells. However, the combination of the two agents resulted in a more substantial increase in the percentage of dead cells (Fig. 1F). Consistently, western blot analysis showed that SNS-032 enhanced the apoptosis-induced by CDDP, as evidenced by Caspase-3 activation and specific cleavage of poly ADP-ribose polymerase (PARP) in KYSE150 and KYSE30 cells (Fig. 1G). We also demonstrated that simultaneously knocking down both CDK7 and CDK9 by lentiviral shRNAs improved the sensitivities of the two tested ESCC cells to CDDP (Fig. 1H). Moreover, the downregulation of Mcl-1 but not matrix metalloproteinase 1 (MMP-1) induced by CDDP was further enhanced after knockdown of both CDK7 and CDK9 (Fig. 1H). We then explored whether SNS-032 synergizes with CDDP to inhibit ESCC cell growth in vivo. As illustrated in Fig. 1I, compared with SNS-032 or CDDP alone treated group, the tumor volume was smaller and the tumor weight was lower in both agents’ treated group. In addition, western blot analysis also showed that the protein level of Mcl-1 was much lower in both SNS-032- and CDDP-treated group than each agent alone (Fig. 1J). Taken together, these findings suggest that the combinational regimen of SNS-032 and CDDP might provide a novel therapeutic approach for the treatment of patients with ESCC.

### SNS-032 induces apoptosis in ESCC cells

**Fig. 2:**
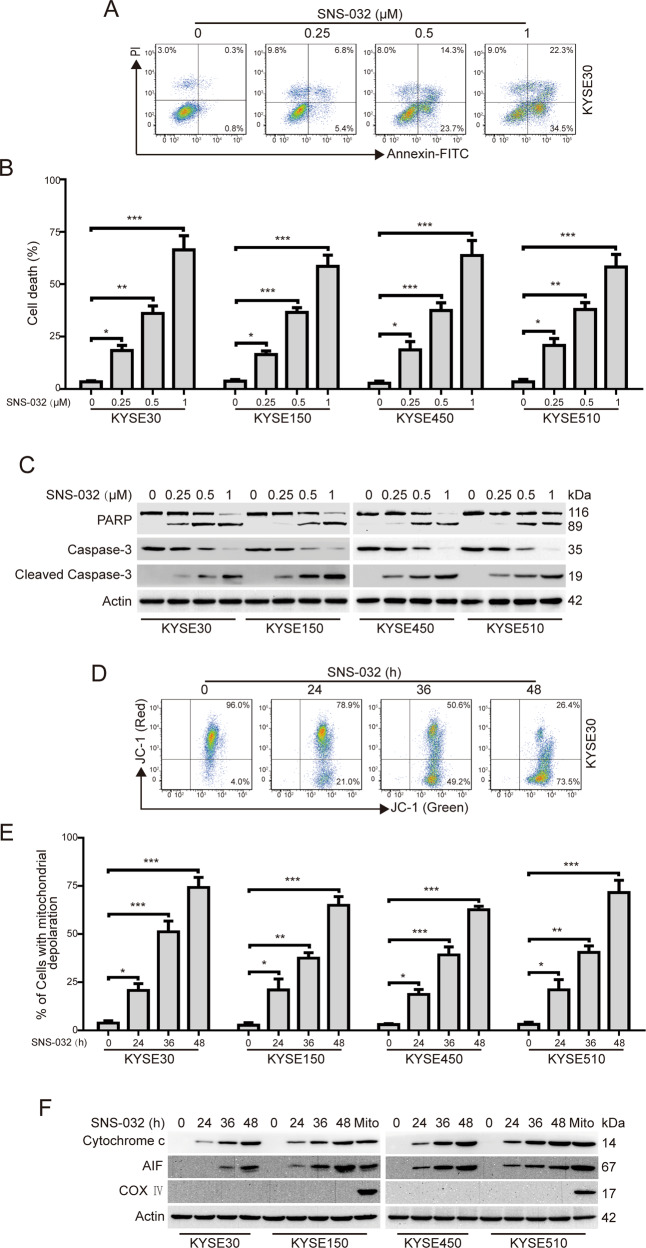
SNS-032 induces apoptosis in ESCC cells. **A**, **B** ESCC cells were incubated with increasing concentrations of SNS-032 for 48 h; the apoptotic cells were detected using flow cytometry after dual staining with annexin V-FITC and PI. **A** Representative flow cytometric dot plots for KYSE30 cells are shown; **B** quantitative analysis of dead cells from five independent experiments. Dead cells were the sum of cells with single or dual stained by annexin V-FITC or PI. C Dose-dependent cleavage of PARP and Caspase-3 was detected by western blot analysis in ESCC cells incubated with SNS-032 for 48 h. **D**, **E** SNS-032 triggered loss of mitochondrial membrane potential in ESCC cells. ESCC cells (KYSE30, KYSE70, KYSE450, and KYSE510) were treated with or without 0.25 μM SNS-032 for the indicated durations, stained with JC-1, and subsequently detected by flow cytometry. **D** Representative flow cytometric dot plots for KYSE30 cells are shown; **E** quantitative analyses from five independent experiments are shown. *P < 0.05, **P < 0.01, ***P < 0.001, one-way ANOVA with post hoc intergroup comparison by Tukey’s test. **F** SNS-032 induced the release of Cytochrome c and AIF from mitochondrial into cytosol in ESCC cells. Mito (mitochondria): the positive control; COX IV served as a mitochondrial indicator to rule out contamination of cytosolic fractions from mitochondria. Actin was used as an internal control.

We next explored whether SNS-032 induces apoptosis in ESCC cells. The ESCC cells were pretreated with increasing doses of SNS-032 for 48 h; apoptosis was then evaluated by flow cytometry after staining with annexin V–fluorescein isothiocyanate (FITC) and propidium iodide. The results showed that SNS-032 treatment elicited a remarkable apoptotic cell death in a concentration-dependent manner in all the four tested ESCC lines (Fig. 2A, B). In addition, western blot analysis also showed that SNS-032 exposure led to an obvious increase of the cleaved PARP and activation of Caspase-3, whereas the protein levels of pro-Caspase-3 was decreased (Fig. 2C), further confirming that SNS-032 induced apoptosis in ESCC cells. To further determine the underlying molecular mechanism of SNS-032-induced apoptosis, ESCC cells incubated with SNS-032 (0.25 μM) for various durations, were stained with JC-1, and then were subjected to flow cytometric analysis. The results indicated that SNS-032 treatment led to a time-dependent increase of cell population with loss of ΔΨm (Fig. 2D, E), which was consistent with other apoptotic indices including activation of Caspase-3 and cleavage of PARP in Fig. 2C. In addition, immunoblotting analysis also showed that the protein levels of apoptosis-inducing factor 1 (AIF) and Cytochrome c in the cytosolic fractionations were much higher in SNS-032-treated ESCC cells than in those of the control group (Fig. 2F). In conclusion, these findings indicated that SNS-032 induces apoptosis of ESCC cells via the mitochondrial (intrinsic) signaling pathway.

### Mcl-1 plays a critical role in SNS-032-induced apoptosis in ESCC cells

**Fig. 3:**
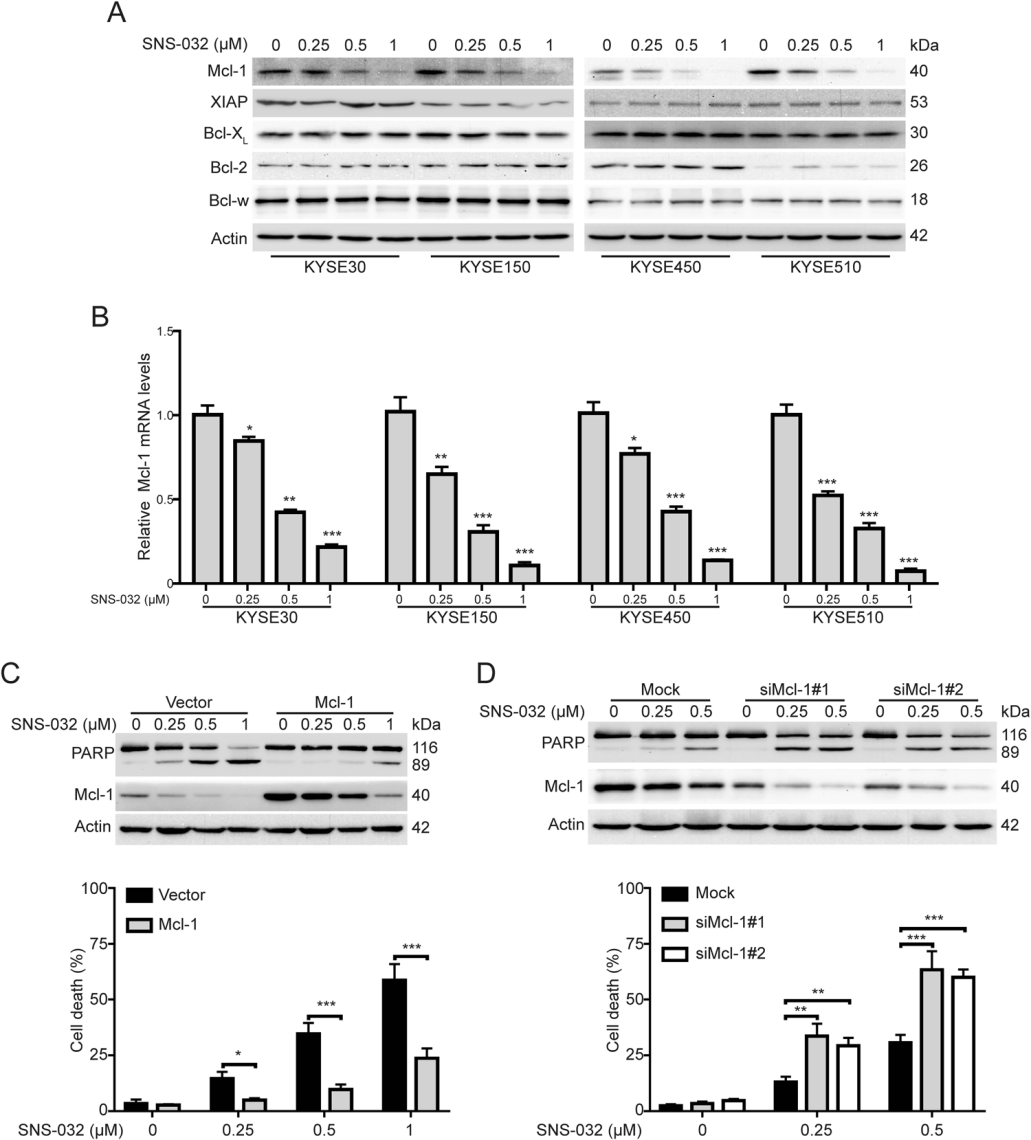
Mcl-1 plays a critical role in SNS-032-induced apoptosis in ESCC cells. **A** Western blot analysis of the apoptosis-related protein expression in ESCC cells treated with SNS-032. **B** SNS-032 dose-dependently decreased mRNA levels of Mcl-1 in ESCC cells. **C** Overexpression of Mcl-1 abrogated the apoptosis induced by SNS-032. After transfection with the indicated plasmids (empty vector and Mcl-1) for 48 h, KYSE30 cells were treated with SNS-032 for 48 h, and then were detected by western blot analysis (top) and trypan blue exclusion assay. **D** Knocking down Mcl-1 sensitized the SNS-032-induced apoptosis. After transduction with the indicated siRNA (Mock, siMcl-1#1 and siMcl-1#2) for 48 h, KYSE30 cells were treated with SNS-032 for 48 h, and then were detected by western blot analysis (top) and trypan blue exclusion assay (bottom). *P < 0.05; **P < 0.01; ***P < 0.001, one-way ANOVA, post hoc intergroup comparisons, Tukey’s test.

It is well known that Bcl-2 family proteins play a crucial role in regulating the intrinsic apoptosis pathway. We therefore examined whether SNS-032 influenced the protein levels of Bcl-2 family members (XIAP, Mcl-1, Bcl-X_L_, Bcl-2, and Bcl-w) using immunoblotting analysis. The results showed that the expression of Bcl-X_L_, Bcl-2, and Bcl-w were not altered in response to SNS-032 treatment. The protein level of XIAP was just slightly reduced in KYSE150 cells but not in the other three tested ESCC cell lines upon SNS-032 treatment. Intriguingly, SNS-032 drastically decreased the protein level of Mcl-1 (Fig. 3A). In reverse transcription–quantitative polymerase chain reaction (RT-qPCR) assay, we found that SNS-032 treatment resulted in a dose-dependent decrease of Mcl-1 mRNA level (Fig. 3B), indicating that SNS-032 transcriptionally downregulates Mcl-1 expression. To further confirm the importance of Mcl-1 in SNS-032-induced apoptosis in ESCC cells, KYSE30 cells transfected with a plasmid encoding human Mcl-1 were incubated with SNS-032 for 48 h, and the influence of ectopic expression of Mcl-1 on SNS-032-mediated apoptosis was examined by western blotting and trypan blue exclusion assay. As illustrated in Fig. 3B, the expression of cleavage of PARP and the percentage of dead cells were notably attenuated in KYSE30 cells with Mcl-1 overexpression, compared to those of the control ones. In contrast, knocking down Mcl-1 by small interfering RNAs (siRNAs) potentiated the apoptosis of KYSE30 cells induced by SNS-032, as evidence by higher protein levels of PARP cleavage and larger percentage of dead cells in Mcl-1-depleted cells than those of tumor cells transfected with Mock siRNA (Fig. 3D). Altogether, these data confirmed that Mcl-1 played a critical role in SNS-032-induced apoptosis in ESCC cells.

### SNS-032 suppresses migration and invasion of ESCC cells

**Fig. 4:**
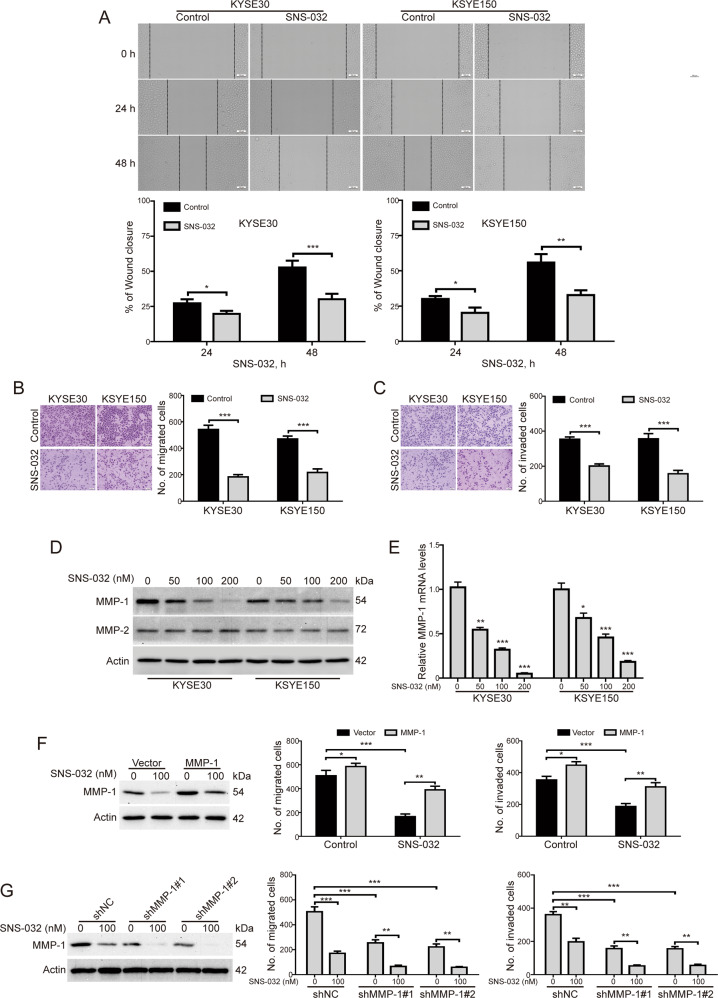
SNS-032 attenuated migration and invasion of ESCC cells. **A** Wound healing assay was performed to examine the migration of ESCC cells (KYSE30 and KYSE150) incubated with 100 nM SNS-032. Scale bar: 100 μm. *P < 0.05, **P < 0.01, ***P < 0.001, by Student’s t test. **B**, **C** SNS-032 suppressed the migration (**B**) and invasion (**C**) of ESCC cells detected by transwell assays. Left: Representative images; Right: Quantitative analysis of five independent experiments. Scale bar: 100 μm. ***P < 0.001, by Student’s *t* test. **D** Western blot analysis of MMP-1 and MMP-2 in KYSE30 and KYSE150 cells treated with increasing concentrations of SNS-032 for 48 h. **E** SNS-032 dose-dependently decreased mRNA levels of MMP-1 in ESCC cells. *P < 0.05; **P < 0.01; ***P < 0.001, one-way ANOVA, post hoc intergroup comparisons, Tukey’s test. **F** Enforced overexpression of MMP-1 rescued the inhibition of migration and invasion by SNS-032 in KYSE30 cells. *P < 0.05; **P < 0.01; ***P < 0.001, one-way ANOVA, post hoc intergroup comparisons, Tukey’s test. **G** Silencing MMP-1 by lentiviral shRNA significantly enhanced the inhibitory effects of SNS-032 on the migration and invasion of KYSE30 cells. **P < 0.01; ***P < 0.001, one-way ANOVA, post hoc intergroup comparisons, Tukey’s test.

Next, we explored whether SNS-032 inhibits the migration of ESCC cells. As shown in Fig. 4A, the migration of KYSE30 and KYSE150 cells was remarkably inhibited after treatment with SNS-032 (100 nM) for 24 h; the inhibition was further upregulated by SNS-032 at 48 h (Fig. 4A). Consistently, transwell migration assay also showed that SNS-032 effectively decreased the number of cells migrated through the membrane in both the tested cell lines (Fig. 4B). We also examined whether SNS-032 suppresses invasion of ESCC cells by using transwell invasion assay. Interestingly, upon SNS-032 treatment, the invasion of KYSE30 and KYSE150 cells was obviously inhibited, compared with the control groups, respectively (Fig. 4C). We next investigated whether SNS-032 suppresses the expression of MMP-1 and MMP-2, two critical metastasis-associated proteins in ESCC cells. The results showed that the protein levels of MMP-1, but not MMP-2, were drastically downregulated in a dose-dependent manner upon SNS-032 treatment in both the tested ESCC cells (Fig. 4D). In RT-qPCR assay, we found that SNS-032 treatment resulted in a dose-dependent decrease of the mRNA level of MMP-1, indicating that SNS-032 transcriptionally downregulates MMP-1 expression (Fig. 4E). To further explore whether MMP-1 was involved in the blockage of migration and invasion induced by SNS-032, KYSE30 cells was stably transduced with a plasmid encoding human MMP-1 and then exposed to 100 nM of SNS-032 and subsequently subjected to Boyden chamber transwell assay. The results showed that forced overexpression of MMP-1 significantly rescued the inhibition of migration and invasion by SNS-032 (Fig. 4F). Conversely, knocking down MMP-1 by lentiviral shRNAs greatly enhanced the SNS-032-mediated decrease in migration and invasion of KYSE30 cells (Fig. 4G). Taken together, these data suggest that SNS-032 effectively inhibits the migration and invasion of ESCC cells through transcriptional downregulation of MMP-1.

### SNS-032 inhibits ESCC cell growth in nude mice

**Fig. 5:**
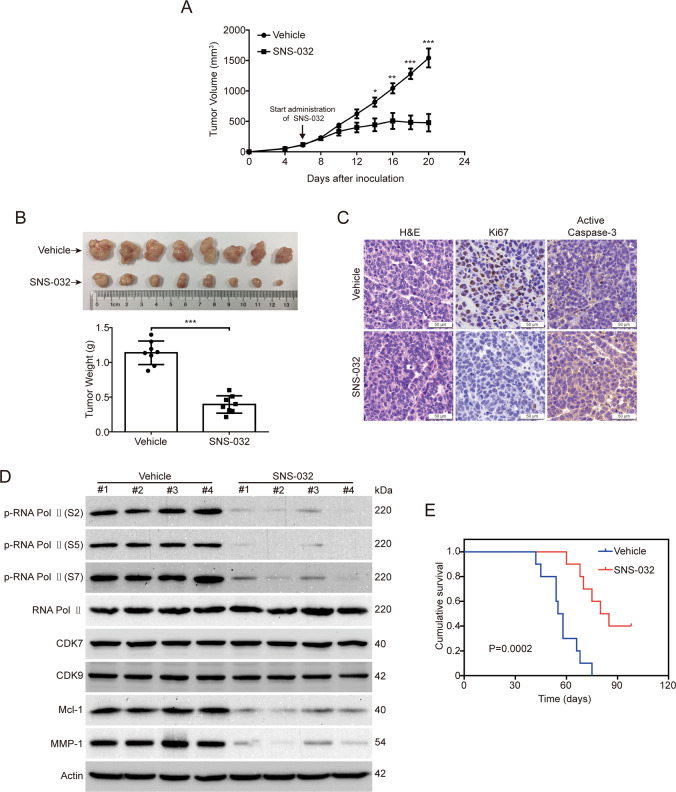
SNS-032 diminished the outgrowth of ESCC cells in nude mice. **A** The tumor size measured at the indicated time point versus time was plotted. *P < 0.05, **P < 0.01, ***P < 0.001, by Student’s t test. **B** Representative images (left panel) and the quantitative analysis (right panel) of isolated tumors derived from mice of each group are shown. ***P < 0.001, by Student’s t test. **C** H&E and IHC staining of xenograft tissues with anti-Active Caspase-3 (apoptosis marker) and anti-Ki67 (proliferation marker) from vehicle- or SNS-032-treated mice are shown. Scale bar: 50 μm. **D** Western blot analysis of the indicated proteins in tumor sections from vehicle- or SNS-032-treated mice. **E** An increase in the life span was observed in the SNS-032-treated group compared with the control group (*n* = 10).

Given that the in vitro antitumor effect of SNS-032, we then examined whether SNS-032 inhibits ESCC cell growth in vivo by using nude mice bearing KYSE30 xenografts. When the tumor xenografts were grown to approximately 100 mm^3^, the mice were randomly divided into two groups (*n* = 8 per group) and received treatment with SNS-032 (15 mg/kg/day) or vehicle for 14 days. As shown in Fig. 5A, the tumor growth curve (tumor volume versus time) was much lower in the SNS-032-treated group, compared with than the vehicle-treated control ones. Additionally, the tumor size and the tumor weight were significantly impaired by SNS-032, in comparison with the vehicle-treated group (Fig. 5B). Importantly, there was no obvious side effect during the treatment (data not shown). Next, we ascertained whether SNS-032 inhibits cell proliferation and induces apoptosis of ESCC cells in vivo by using IHC staining. The results showed that the proliferation (marked as Ki67 staining) of ESCC cells was drastically diminished upon SNS-032 administration (Fig. 5C). In addition, the apoptosis (marked as Active Caspase-3 staining) of ESCC cells was obviously increased in tumor tissues of the SNS-032-treated group (Fig. 5C). We then examined whether SNS-032 restricts the phosphorylation status of RNA Pol II by using western blot analysis. As expected, the CTD phosphorylation of RNA Pol II at Ser 2, 5, and 7 sites in tumor tissues was significantly dampened upon SNS-032 treatment (Fig. 5D). Moreover, the protein levels of Mcl-1 and MMP-1 were also dramatically downregulated by SNS-032, compared to the control group (Fig. 5D), which was in line with the in vitro findings. More importantly, SNS-032 treatment significantly increased the overall survival of KYSE30 cell-bearing mice, compared with the vehicle-treated group (Fig. 5E). Taken together, these results demonstrated that SNS-032 inhibits the growth of ESCC cells in vivo.

### SNS-032 diminishes ESCC cell metastasis in nude mice

**Fig. 6:**
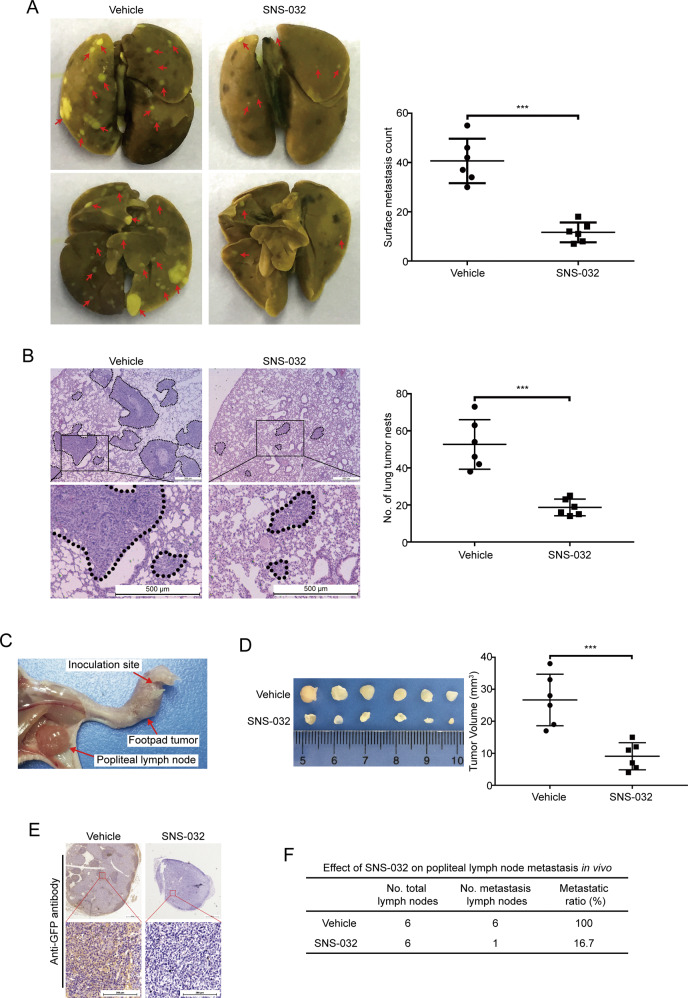
SNS-032 suppresses lung and lymphatic metastasis of ESCC cells in vivo. **A**, **B** BALB/c nude mice were intravenously injected with KYSE30 cells via the lateral tail vein and administered with vehicle or SNS-032 for 2 weeks. **A** Representative micrographs of lungs (left) and the statistical analysis of surface metastatic nodules (right) in vehicle- or SNS-032-treated mice are shown. Arrows indicate the metastatic tumors. **B** H&E-stained sections of representative lungs (left) and the quantitative analysis of metastatic tumors (right) from each group are shown. Scale bar: 500 μm. **C**–**F** SNS-032 mitigates lymph node metastasis in vivo. A popliteal lymph node metastasis model was established by inoculating KSYE30 cells stably expressing GFP into the foot pads of nude mice. **C** The foot pad tumors and popliteal lymph nodes were enucleated and analyzed 6 weeks after injection of KYSE30 cells. **D** Representative images (left) and the volumes of popliteal lymph nodes (right). **E** Representative images of the popliteal lymph nodes immunostained with anti-GFP antibody. Scale bars: 200 μm. **F** The ratios of metastatic to total dissected popliteal lymph nodes from mice of each group. ***P < 0.001, by Student’s *t* test.

Considering that SNS-032 could effectively inhibit ESCC cell migration and invasion in vitro, a lung metastasis mouse model by intravenously injecting KYSE30 cells was performed to investigate whether SNS-032 inhibits ESCC metastasis in vivo. As shown in Fig. 6A, the results showed that SNS-032 treatment resulted in an obvious decrease the number of metastatic nudes and the tumor size on the lung surface of each mouse, compared to that derived from vehicle-treated mice. Additionally, hematoxylin and eosin (H&E) staining of lung tissues also showed an obviously smaller tumor size and fewer number of metastatic nudes in the lungs of SNS-032-treated mice, compared with those of the vehicle-treated mice (Fig. 6B).

To further determine the inhibitory effects of SNS-032 on ESCC metastasis, an in vivo popliteal lymph node metastasis model was employed in nude mice by inoculating the foot pads with KYSE30 cells stably expressing green fluorescent protein (GFP; Fig. 6C). We found that SNS-032 greatly attenuated lymph node metastasis, as evidenced by smaller volumes of lymph nodes and decreased numbers of GFP-positive tumor cells in tumors formed from SNS-032-treated mice, compared with those of the vehicle-treated mice (Fig. 6D, E). Strikingly, the ratios of metastatic to total dissected popliteal lymph nodes were markedly lower in mice from the SNS-032-treated group (100% (6/6)) than in the vehicle controls (16.7% (1/6); Fig. 6F).

Taken together, these results demonstrate that SNS-032 effectively abrogates the metastasis of ESCC cells in nude mice.

## Discussion

In this study, we discovered that both CDK7 and CDK9 are highly elevated in all five tested human ESCC cells, suggesting existence of elevated transcription activity. Transcription inhibition by SNS-032 significantly suppressed the cellular proliferation, anchorage-independent growth, and the growth of xenografted ESCC cells in nude mice. Moreover, SNS-032 drastically inhibited the migration and invasion of ESCC cells by reducing the transcription of MMP-1. Most importantly, SNS-032 profoundly diminished the lung and lymph node metastasis of ESCC cells in vivo. Our studies provided a new strategy for the treatment of ESCC by targeting transcription of the oncogene.

As a specific CDK inhibitor against CDK7 and CDK9, SNS-032 has displayed potent anticancer effects in various human malignant cells, such as uveal melanoma [5], acute myeloid leukemia [24], and cervical cancer cells [25], and exerted chemopreventive effects in both NOD-SCID mouse xenograft model and NOG mouse model of uveal melanoma [5]. In this study, our results showed that SNS-032 significantly inhibited tumor cell growth and induced apoptosis in all the investigated cell lines of ESCC at nanomolar concentrations. Consistently, a phase I clinical trial in patients with metastatic refractory solid tumors showed that the therapeutic SNS-032 plasma concentrations were <754 nM [22], which can be extrapolated to kill ESCC cells. Intriguingly, our results also showed a notable synergistic antiproliferative effect between SNS-032 and CDDP, the first-line chemotherapy for patients with locally advanced ESCC [26]. In agreement with our findings, a few reports have showed that there is a synergistic effect to inhibit malignant cell growth when SNS-032 combined with radiation in cervical cancer and non-small cell lung cancer [25, 27]. Altogether, these findings suggest that SNS-032 in combination with the traditional chemotherapeutic agents is a promising therapeutic strategy for cancer patients. Thus, future investigations are needed to further evaluate the efficiency of SNS-032 together with CDDP in mouse models and clinical trials of ESCC.

In this study, we also identified that Mcl-1 played an important role in SNS-032-induced apoptosis. Mcl-1 belongs to an antiapoptotic protein of the Bcl-2 family members and is mainly localized to the outer mitochondrial membrane [28]. By interaction with the proapoptotic proteins such as Bak or Bax, Mcl-1 is able to disrupt the mitochondrial membrane and ultimately prevents apoptosis [17]. Mcl-1 is frequently overexpressed in various cancers including ESCC [29, 30], and it protects tumor cells from apoptosis induced by chemotherapeutic agents, such as cytarabine, daunorubicin, and regorafenib [31, 32]. Knockdown of Mcl-1 by shRNA or selective inhibitor UMI-77 greatly enhances the sensitivity of ESCC cells to CDDP [33]. In this study, we discovered that SNS-032 treatment led to transcriptional downregulation of Mcl-1 in ESCC cells. In addition, KYSE30 cells transfected with a plasmid encoding human Mcl-1 were resistant to SNS-032-induced apoptosis; in contrast, knockdown of Mcl-1 by lentiviral shRNAs potentiated the apoptosis of ESCC cells induced by SNS-032. These data support a crucial role of Mcl-1 in SNS-032-induced apoptosis in ESCC cells.

Tumor metastasis is one of the most detrimental events that attribute a poor prognosis to ESCC patients, even after effective curative treatment [3, 34]. A 10-year population-based retrospective cohort study of 6102 ESCC cases conducted with the Surveillance, Epidemiology, and End Results Program database showed that ESCC typically metastasized to the lung (15.84%) and distant lymph nodes (15.37%) [35]. In this study, we investigated whether SNS-032 inhibits ESCC metastasis by using a lung metastasis model and a popliteal lymph node metastasis model, respectively. Strikingly, our results demonstrated that SNS-032 remarkably suppressed both the lung and lymph node metastasis in ESCC. Consistent with our findings, Kang et al. reported that SNS‑032 inhibited migration, invasion, and angiogenesis in vitro and suppressed lung metastases in a spontaneous metastasis model in cervical cancer [25]. Additionally, Zhang and colleagues demonstrated that SNS-032 reduced the liver metastasis of uveal melanoma by using a NOG mouse model [5]. Altogether, these findings from us and other colleagues suggest that the CDK7/9 inhibitor SNS-032 is a promising therapeutic agent for the treatment of metastatic cancer patients.

MMPs, as a family of zinc-dependent proteases, play an essential role in facilitating the invasion and metastasis of malignant tumor cells into other organs or tissues by promoting the degradation and proteolysis process of the extracellular matrix [36]. MMP-1 is an important family member of MMPs. Accumulating evidence shows that aberrant expression of MMP-1 has been implicated in the progression of various human cancers, such as colorectal cancer [37], lung adenocarcinoma [38] and ESCC [39]. High expression levels of MMP-1 positively corrected with lymph node metastasis and advanced tumor, node, metastasis stage [40]. Additionally, MMP-1 was considered as an independent factor for overall survival in two independent cohorts of 613 ESCC patients [41]. Functionally, ectopic expression of MMP-1 promoted ESCC cell migration, invasion, and metastasis through the activation of the phosphoinositide-3 kinase/AKT pathway [41]. MMP1 has been shown to function as a prognostic biomarker and a potential therapeutic target in ESCC [41]. In this study, we confirmed that SNS-032 inhibited migration and invasion of ESCC cells via transcriptional inhibition of MMP-1, which was based on the following results: (1) Both the mRNA and protein levels of MMP-1 in ESCC cells were downregulated upon treatment with SNS-032 in a concentration-dependent manner. (2) Ectopic expression of MMP-1 significantly attenuated the inhibitory effects on SNS-032 on the migration and invasion of ESC cells. (3) Silencing MMP-1 by lentiviral shRNAs remarkably enhanced the SNS-032-mediated decrease in migration and invasion of the tested ESCC cells.

In conclusion, we demonstrated that SNS-032, as a potent and selective CDK7/9 inhibitor, which leads to inhibition of transcription initiation and elongation, effectively inhibited cell growth and induced apoptosis in ESCC cells. SNS-032 also exhibits a synergistic effect in inducing cell apoptosis and inhibiting tumor growth when combined with CDDP, a first-line therapeutic agent for ESCC patients. Importantly, SNS-032 potently abrogated migration and invasion in vitro and diminished the lung and lymph node metastasis in vivo. Therefore, blocking transcription by CDK7 and CDK9 inhibitor SNS-032 is a promising strategy to treat ESCC and warrants further clinical investigation to assess its value in patients with metastatic ESCC.

## Materials and methods

### Cell culture

Human ESCC cell lines KYSE30, KYSE150, KYSE450, KYSE510, and TE-1 purchased from the Cell Bank of the Chinese Academy of Sciences (Shanghai, China) were cultured in RPMI 1640 medium (Gibco, USA) containing 10% fetal bovine serum (FBS; Gibco, USA) as previously described [42]. The immortalized human esophageal epithelial cell line Het-1A and human embryonic kidney 293T cells, obtained from the ATCC (Manassas, VA, USA), were cultured in Dulbecco’s modified Eagle’s medium culture medium supplemented with 10% FBS. Cells were kept in a humidified incubator with 5% CO_2_ at 37 °C. All cells were periodically authenticated by using the short tandem repeat analysis and tested for mycoplasma contamination by PCR.

### Reagents and antibodies

SNS-032 (#S1145) and CDDP (#S1166) were purchased from Selleck Chemicals (Shanghai, China). The antibodies against RNA Pol II (#A300-653A), p-RNA Pol II (S5) (#A304-408A), and p-RNA Pol II (S2) (#A300-654A) were obtained from Bethyl Laboratories (Montgomery, TX). The anti-p-RNA Pol II (S7) (#04-1570) was purchased from EMD Millipore (Billerica, MA). The anti-CDK7 antibody (#2916), anti-CDK9 antibody (#2316), anti-Caspase-3 antibody (#9665), anti-PARP antibody (#9532), anti-Cleaved Caspase-3 antibody (#9664), anti-Bcl-X_L_ antibody (#2762), anti-Bcl-w (31H4) (#2724), anti-XIAP antibody (#2042), anti-Mcl-1 antibody (#5453), anti-Cytochrome c antibody (#4272), anti-AIF antibody (#5318), anti-COX IV antibody (#4850), anti-MMP-2 antibody (#4022), and anti-GFP antibody (#2956) were obtained from Cell Signaling Technology (CST, Danvers, MA, USA). The antibody against Ki67 (#ab15580) and MMP-1 (#ab137332) were obtained from Abcam (Cambridge, UK). The anti-Actin antibody (#4700) was purchased from Sigma (St. Louis, MO, USA). Horseradish peroxidase (HRP)-conjugated secondary antibodies including Goat anti-Mouse IgG (#CW0102S) and Goat anti-Rabbit IgG (#CW0103S) were bought from CWBio (Beijing, China).

### Quantitative real-time PCR (qRT-PCR)

Total mRNAs were isolated by using the TRIzol reagent (Invitrogen, Shanghai, China) according to the manufacturer’s instructions. RT was carried out by using the PrimeScript™ RT Master Mix (Perfect Real Time) (TaKaRa, Dalian, China). qRT-PCR reactions were performed on the LightCycler^®^ 480 system (Roche Diagnostics, Risch, Switzerland) by using LightCycler^®^ 480 SYBR Green I Master (Roche Diagnostics, Risch, Switzerland). The primers used in this study are listed in Supplementary Table S1. Glyceraldehyde-3-phosphate dehydrogenase was used as the internal control gene and the fold enrichment was calculated using the 2^−ΔΔCt^ method.

### Cell viability assay

MTT assay was performed to assess the cell viability of ESCC cells. Briefly, 2000 cells were plated in 96-well plates (Corning). Twenty-four hours later, escalating concentrations of SNS-032 were added. Four hours before the end of experiments, 20 μl of MTT reagent (Sigma-Aldrich, USA) was added to each well. At last, 150 μl of dimethyl sulfoxide (DMSO) was added to dissolve the formazan. Absorbance was recorded by using a Synergy HT Microplate Reader (BioTek, Winooski, VT, USA) at a wavelength of 570 nm. IC_50_ values were calculated by using GraphPad Prism version 7.0 (GraphPad Software, San Diego, CA).

### Soft agar assay

The anchorage-independent growth was determined by using double layer soft agar system as our described previously [43]. Briefly, ESCC cells were cultured with increasing concentrations of SNS-032 for 48 h; cells that were then collected and resuspended in complete RPMI1640 medium (500 μl) containing 0.4% agar were plated over the layer of 0.8% agar containing medium in 24-well plates (2000 cells per well) in triplicates and incubated in a humidified incubator at 37 °C for 2 weeks. Colonies containing >50 cells were counted with an inverted optical microscope.

### Cell apoptosis by flow cytometry

Cells were collected and stained with an annexin V-FITC Apoptosis Detection Kit (Sigma-Aldrich, Shanghai, China) according to the manufacturer’s instructions. The apoptotic cells were immediately detected and analyzed by using the BD FACSVerse flow cytometry and its software (BD Biosciences) as previously described [43].

### Evaluation of mitochondrial membrane potential (ΔΨm)

The ΔΨm was measured as previously described [44]. In brief, ESCC cells were pretreated with increasing concentrations of SNS-032 for 24 h; cells were then harvested and incubated with a ΔΨm assay kit with JC-1 (Beyotime, Shanghai, China) at 37 °C for 30 min. After washing with phosphate-buffered saline (PBS), cells were subjected to flow cytometric analysis for the ΔΨm.

### Western blot analysis

Cytosolic fractionations for detection AIF and Cytochrome c were prepared in digitonin extraction buffer (0.015% digitonin, 300 mM sucrose, 10 mM PIPES, 3 mM MgCl_2_, 100 mM NaCl, 5 mM EDTA) supplemented with 1 mM phenylmetnylsulfonyl fluoride (PMSF). The whole-cell lysates were prepared in RIPA buffer (1× PBS, 0.5% sodium deoxycholate, 1% NP-40, and 0.1% sodium dodecyl sulfate (SDS)) containing 10 mM NaF, 10 mM β-glycerophosphate, 1 mM PMSF and protease inhibitor cocktail (Roche, Indianapolis, IN). Protein concentrations were detected with the Pierce BCA Protein Assay Kit (#23222, Thermo Scientific) according to the manufacturer’s instructions. Thirty μg of total proteins were separated by using SDS–polyacrylamide gel electrophoresis and transferred onto nitrocellulose membranes. After incubation with 5% dried skimmed milk, the NC membranes were incubated with the indicated primary antibodies at 4 °C overnight and then treated with the corresponding HRP-linked secondary antibodies at room temperature for 2 h. The protein bands were developed by using enhanced chemiluminescence (ECL) detection reagent (Beyotime, Shanghai, China) and then scanned using the Amersham ImageQuant 800 system (GE Healthcare, Piscataway, USA).

### Wound healing assay

ESCC cells (KYSE30 and KYSE150) were plated into a 6-well flat-bottom plate (Corning) and incubated overnight to growth to confluence cell monolayer. After scratching with a 200 μl pipette tip, the plates were washed with 1× PBS to remove the floating cells and added fresh serum-free RPMI1640 medium. The cells were incubated with SNS-032 and captured by using an inverted phase-contrast microscope at the indicated time point post-scratching. The migration rate was calculated by the formula: (gap distance at 0 h − gap distance at the indicated time)/(gap distance at 0 h) × 100% [45].

### Cell migration and invasion assays

The migration and invasion assays were conducted by using 24-well plates with polycarbonate sterile membrane (8 μm pore size, Corning, NY, USA) as previously described [46]. For migration assay, ESCC cells (2.5 × 10^5^/ml) pretreated with SNS-032 were resuspended in serum-free RPMI1640 medium (200 μl) and seeded into the upper chamber of transwell inserts. Medium (600 μl) containing 10% FBS was added to the lower chamber. After incubation at 37 °C for 24 h, the transwell inserts were treated with 4.0% paraformaldehyde solution and then stained with 0.1% crystal violet. The cells inside the upper chambers were removed with a cotton swab. The cells attached to the lower surface were recorded using an inverted phase-contrast microscope. The transwell invasion assay was performed using a similar protocol, except that the tumor cells (5 × 10^5^/ml) were added to the upper chamber of inserts embedded with Matrigel (BD Biosciences, CA).

### Transfection of plasmids and lentivirus transduction

The full-length human Mcl-1 and MMP-1 cDNA were cloned into the pCMV-Flag-His-puro vector by Transheep (Shanghai, China). For overexpression experiment, 2 μg plasmids (empty vector/Mcl-1/MMP-1) were transfected into the indicated tumor cells by using the Lipofectamine 2000 reagent (Invitrogen, Thermo Fisher Scientific, Inc.) according to the manufacturer’s manual. Forty-eight hours after transfection, the cells were screened with 1.5 μg/ml puromycin (Sigma, St. Louis, USA) for 2 weeks.

shRNA sequences (Supplementary Table S2) were cloned into the pLKO.1-puro vector (Sigma, St. Louis, USA). The target lentiviral constructs or control together with pMD2.G (#12259, Addgene) and psPAX2 (#12260, Addgene) were transduced in 293T cells using PEI reagent (Polysciences, Warrington, USA). The lentivirus productions collected at 72 h after transfection were purified using 0.45-μm filters and subsequently were added to the indicated tumor cells supplemented with 8 μg/ml polybrene (Millipore, Burlington, MA, USA). The transduced cells were selected in the presence of 1.5 μg/ml puromycin (Sigma, St. Louis, USA) for 14 days.

### RNA interference

Mcl-1 siRNAs and scramble siRNA (Mock) were purchased from Sigma-Aldrich (Shanghai, China). To achieve knockdown of Mcl-1 gene by siRNA, ESCC cells were seeded into a 6-well flat-bottom plate and incubated overnight. Cells were then transfected with 25 nM Mcl-1 siRNAs (siMcl-1#1 and siMcl-1#2) or scramble siRNA (Mock) by using Lipofectamine RNAiMAX transfection reagent (Invitrogen, Shanghai, China) according to the manufacturer’s protocol. Briefly, Lipofectamine RNAiMAX and siRNA were separately diluted in Opti-MEM (Life Technologies, Shanghai, China). The two dilutions were then gently mixed and incubated for 20 min at room temperature. Eight hours after transfection, each well was exchanged with fresh medium.

### Animal experiments

All experimental procedures were approved by the Henan University Institutional Animal Care and Use Committee. Male BALB/c nude mice (5–6 weeks old, 18–20 g) purchased from Vital River (Beijing, China) were bred under specific pathogen-free conditions with a lighting cycle of 12 h darkness/12 h light and controlled temperature (20 ± 2 °C) and humidity (40–50%).

For the in vivo xenograft experiment, KYSE30 cells (5 × 10^6^ cells/mouse) were subcutaneously inoculated into the left dorsal flank of BALB/c nude mice [46]. The tumors were examined with calipers every 2 days and calculated by using the following formula: tumor volume = *L* × *W*^2^ × 0.5, where *L* is the longest diameter and *W* is the diameter perpendicular to *L*. When tumor volume was about 100 mm^3^, the animals were randomly split into two groups (*n* = 8 per group) and intraperitoneally (i.p.) injected with SNS-032 (15 mg/kg/day) or vehicle (PBS with DMSO <0.1%) on a 3-day-on/2-day-off schedule [5]. Fourteen days later, all mice were anesthetized by using isoflurane, and tumor xenografts were harvested, photographed, and weighed. Tumor tissues were prepared cell lysates for western blot analysis or fixed with formalin for IHC and H&E staining.

To investigate whether there was a synergistic effect between SNS-032 and CDDP in inhibiting ESCC growth in vivo, KYSE30 cells (1 × 10^6^ cells/mouse) were subcutaneously inoculated into the left dorsal flank of BALB/c nude mice, When tumor volume was about 100 mm^3^, the animals were randomly split into four groups (*n* = 5 per group) as follows: vehicle, SNS-032 (7.5 mg/kg, i.p.) on a 3-day-on/2-day-off schedule, CDDP (5 mg/kg, i.p.) once per week, or SNS-032 + CDDP. Four weeks later, all mice were anesthetized by using isoflurane, and tumor xenografts were harvested, photographed, and weighed. Tumor tissues were prepared from cell lysates for western blot analysis.

For the lung metastasis model, nude mice intravenously injected via lateral tail vein using KYSE30 cells (1 × 10^6^ in 100 μl PBS) were randomly divided into two groups (*n* = six per group) [46]. Twenty-four hours later, the mice were treated with SNS-032 (15 mg/kg/day) or vehicle (PBS with DMSO <0.1%) on a 3-day-on/2-day-off schedule for 2 weeks. All of the mice were anesthetized with isoflurane 8 weeks post injection with tumor cells. Lungs were harvested and fixed in Bouin’s solution (5.0% acetic acid, 75% saturated picric acid, and 25% formaldehyde). The numbers of metastatic colonies on the lung surface of each mouse were counted, and histological evidence of the lung tissues was examined by H&E staining.

To explore the effect of SNS-032 on the overall survival of ESCC tumor-bearing mice, KYSE30 cells (2 × 10^6^) were intravenously injected into each nude mice via lateral tail vein. Twenty-four hours later, the mice were randomly assigned to two groups (*n* = 10) and treated with SNS-032 (15 mg/kg/day) or vehicle for 2 weeks. The survival time of each mouse was recorded.

For the popliteal lymphatic metastasis experiments, KYSE30 cells stably expressing GFP (1 × 10^6^ cells in 50 μl PBS per mouse) were inoculated into the left foot pad of BALB/c nude mice^3^. The animals were randomly divided into two groups (*n* = six per group) and i.p. injected with vehicle (PBS with DMSO <0.1%) or SNS-032 (15 mg/kg/day) on a 3-day-on/2-day-off schedule for 2 weeks. After 6 weeks injection with tumor cells, mice were killed with isoflurane, and the popliteal lymph nodes were collected, photographed, and then fixed with formalin for H&E or IHC staining.

### H&E and IHC staining

For H&E staining, the tissue slides (4 μm) were deparaffinized with xylene, rehydrated in graded ethanol, and then stained with a Hematoxylin and Eosin Staining Kit (Beyotime, Shanghai, China) according to the manufacturer’s manual. IHC staining was performed as previously described [45]. Briefly, after fixation with formalin, the paraffin-embedded tissue sections (4 μm) were deparaffinized, rehydrated, incubated with hydrogen peroxide (3%), and then retrieved in citrate solution (10 mM, pH 6.0). The slides were blocked with 5% bovine serum albumin and incubated with the indicated primary antibodies at 4 °C for overnight. Color was developed by using a DAB Kit (CWBIO Co., Beijing, China) following the instructions and then counterstained with hematoxylin. Representative images were captured by using an inverted fluorescence microscope (Leica, Germany).

### Statistical analysis

All experiments were performed at least three times and the statistical analysis was conducted by using GraphPad Prism version 7.0 (GraphPad Software, San Diego, CA). Student’s *t* test was used to compare the differences between two groups, whereas three and more groups were compared by using one-way analysis of variance with Tukey’s post hoc test. The overall *F* test was significant (*P* < 0.05), and there was no significant variance in homogeneity. All statistical tests were two-sided, and *P* < 0.05 was considered statistically significant.

## Supplementary information

Reproducibility Checklist

Additional file

Supplementary Figure S1

## Data Availability

All data associated with this study are presented in the paper or Supplementary Materials. Additional data or reagents are available from the corresponding author upon reasonable request.
