# Supplementary material for: Transcriptional inhibition by CDK7/9 inhibitor SNS-032 suppresses tumor growth and metastasis in esophageal squamous cell carcinoma
Source: Cell Death Dis. 2021 Nov 5;12(11):1048. doi: 10.1038/s41419-021-04344-w (PMC8571299; doi:10.1038/s41419-021-04344-w)
Supplement: Supplementary file 2 — Additional file [file 41419_2021_4344_MOESM2_ESM.docx]

**Supplementary Table S1. Primers for qRT-PCR analysis.**

| **Genes** | **Sense primer** | **Antisense primer** |
| --- | --- | --- |
| **Mcl-1** | 5′-TGCTTCGGAAACTGGACATCA-3′ | 5′-TAGCCACAAAGGCACCAAAAG-3′ |
| **MMP-1** | 5′-AAATGCAGGAATTCTTTGGG-3′ | 5′-ATGGTCCACATCTGCTCTTG-3′ |
| **GAPDH** | 5'-GATCGAATTAAACCTTATCGTCGT-3' | 5'-AGCAGCAGAACTTCCACTCGGT-3' |

**Supplementary Table S2. Primers for shRNA.**

| **Gene** | **Sequence (5' to 3')** |
| --- | --- |
| **shNC (No target shRNA)** | CCGGGCGCGATAGCGCTAATAATTTCTCGAGAAATTATTAGCGCTATCGCGCTTTTT |
| **shMMP-1#1** | CCGGTGAAGATGAAAGGTGGACCAACTCGAGTTGGTCCACCTTTCATCTTCATTTTT |
| **shMMP-1#2** | CCGGGCTAACCTTTGATGCTATAACCTCGAGGTTATAGCATCAAAGGTTAGCTTTTTG |
| **shCDK2#1** | CCGGGCCTGATTACAAGCCAAGTTTCTCGAGCGGACTAATGTTCGGTTCAAATTTTT |
| **shCDK2#2** | CCGGCTATGCCTGATTACAAGCCAACTCGAGGATACGGACTAATGTTCGGTTTTTTT |
| **shCDK7#1** | CCGGGCAGGAGACGACTTACTAGATCTCGAGCGTCCTTCTGCTGAATGATCTATTTTT |
| **shCDK7#2** | CCGGTCAGAAGCTAAAGATGGTATACTCGAGAGTCTTCGATTTCTTACCATATTTTTT |
| **shCDK9#1** | CCGGCTACTACATCCACAGAAACAACTCGAGGATGATGTAGGTGTCTTTGTTTTTTT |
| **shCDK9#2** | CCGGTGATTGAGATTTGTCGAACCACTCGAGACTAACTCTAAACAGCTTGGTTTTTT |

**Supplementary Figure S1**. Silencing CDK7 or CDK9 but not CDK2 decreases the sensitivities of ESCC cells to SNS-032. After transduced with shRNA against CDK2 (A), CDK7 (B) or CDK9 (C), KYSE30 and KYSE150 cells were exposed to increasing concentrations of SNS-032 for 72 h, and cell viability was measured by MTT assay.
