# Supplementary figures and images for: Transcriptional inhibition by CDK7/9 inhibitor SNS-032 suppresses tumor growth and metastasis in esophageal squamous cell carcinoma
Source: Cell Death Dis. 2021 Nov 5;12(11):1048. doi: 10.1038/s41419-021-04344-w (PMC8571299; doi:10.1038/s41419-021-04344-w)

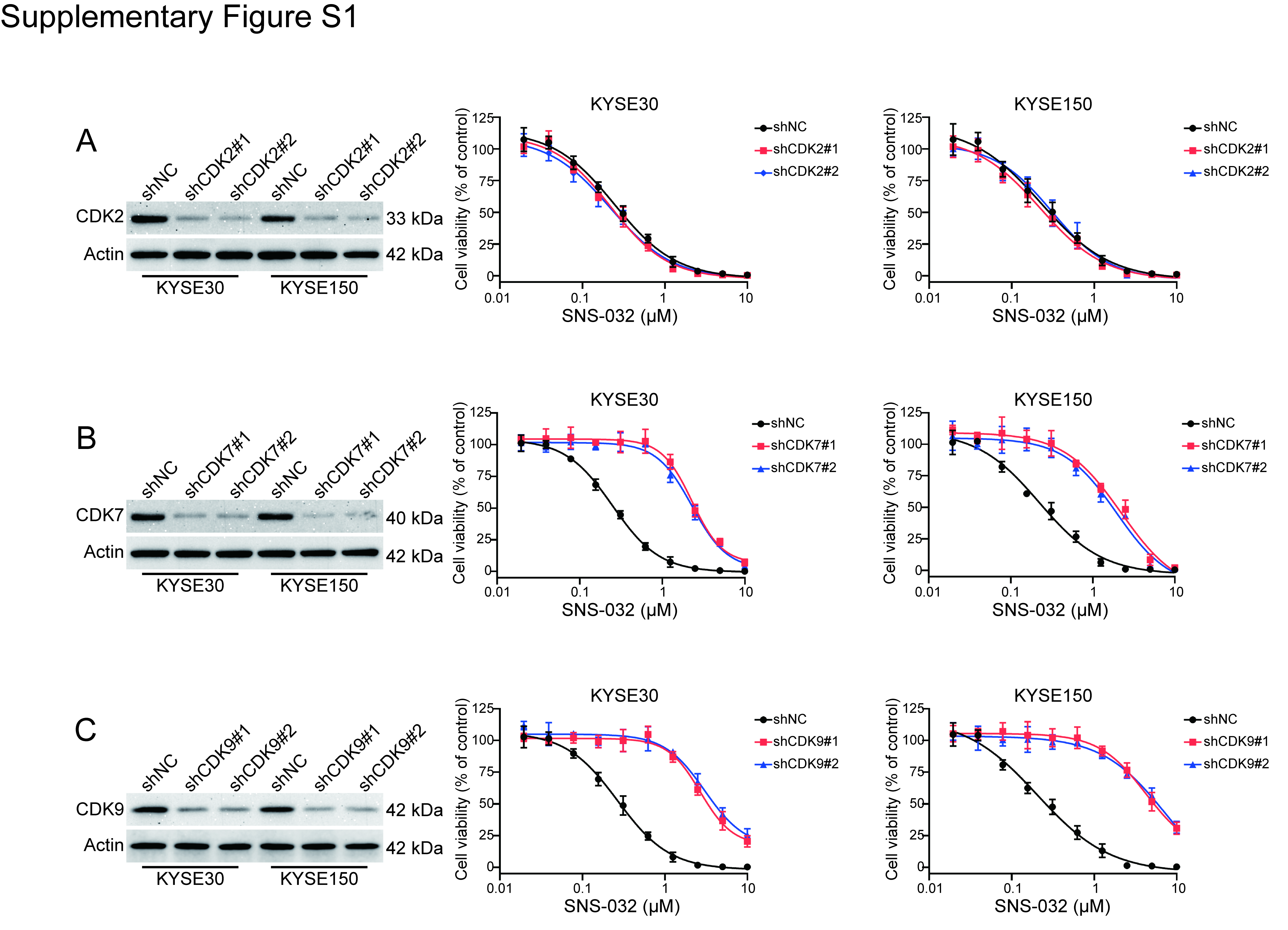

Supplement: Supplementary file 3 — Supplementary Figure S1 [file 41419_2021_4344_MOESM3_ESM.tif]
